# Molecular Pap smear: HPV genotype and DNA methylation of *ADCY8*, *CDH8*, and *ZNF582* as an integrated biomarker for high-grade cervical cytology

**DOI:** 10.1186/s13148-016-0263-9

**Published:** 2016-09-13

**Authors:** Jane Shen-Gunther, Chiou-Miin Wang, Graham M. Poage, Chun-Lin Lin, Luis Perez, Nancy A. Banks, Tim Hui-Ming Huang

**Affiliations:** 1 Gynecologic Oncology & Clinical Investigation, Department of Clinical Investigation, Brooke Army Medical Center, 3698 Chambers Pass, Fort Sam Houston, TX 78234-6315 USA; 2 Department of Molecular Medicine, Cancer Therapy and Research Center, University of Texas Health Science Center at San Antonio, San Antonio, TX 78229 USA; 3 Department of Clinical Investigation, Brooke Army Medical Center, Fort Sam Houston, TX 78234 USA; 4 Department of Pathology and Area Laboratories, Brooke Army Medical Center, Fort Sam Houston, TX 78234 USA

**Keywords:** HPV, HPV genotyping, DNA methylation, Pap smear, Pyrosequencing, Molecular diagnostics, Molecular biomarkers

## Abstract

**Background:**

The Pap smear has remained the foundation for cervical cancer screening for over 70 years. With advancements in molecular diagnostics, primary high-risk human papillomavirus (hrHPV) screening has recently become an accepted stand-alone or co-test with conventional cytology. However, both diagnostic tests have distinct limitations. The aim of this study was to determine the association between HPV genotypes and cellular epigenetic modifications in three grades of cervical cytology for screening biomarker discovery.

**Methods:**

This prospective, cross-sectional study used residual liquid-based cytology samples for HPV genotyping and epigenetic analysis. Extracted DNA was subjected to parallel polymerase chain reactions using three primer sets (MY09/11, FAP59/64, E6-E7 F/B) for HPV DNA amplification. HPV+ samples were genotyped by DNA sequencing. Promoter methylation of four candidate tumor suppressor genes (adenylate cyclase 8 (*ADCY8*), cadherin 8, type 2 (*CDH8*), *MGMT*, and zinc finger protein 582 (*ZNF582*)) out of 48 genes screened was quantified by bisulfite-pyrosequencing of genomic DNA. Independent validation of methylation profiles was performed by analyzing data from cervical cancer cell lines and clinical samples from The Cancer Genome Atlas (TCGA).

**Results:**

Two hundred seventy-seven quality cytology samples were analyzed. HPV was detected in 31/100 (31 %) negative for intraepithelial lesion or malignancy (NILM), 95/100 (95 %) low-grade squamous intraepithelial lesion (LSIL), and 71/77 (92 %) high-grade squamous intraepithelial lesion (HSIL) samples. The proportion of IARC-defined carcinogenic HPV types in sequenced samples correlated with worsening grade: NILM 7/29 (24 %), LSIL 53/92 (58 %), and HSIL 65/70 (93 %). Promoter methylation of *ADCY8*, *CDH8*, and *ZNF582* was measured in 170 samples: NILM (*N* = 33), LSIL (*N* = 70), and HSIL (*N* = 67) also correlated with worsening grade. Similar hypermethylation patterns were found in cancer cell lines and TCGA samples. The combination of four biomarkers, i.e., HPV genotype and three-gene promoter methylation, predicted HSIL (AUC 0.89) better than HPV alone (AUC 0.74) by logistic regression and probabilistic modeling.

**Conclusions:**

HPV genotype and DNA methylation of *ADCY8*, *CDH8*, and *ZNF582* are correlated with cytological grade. Collectively, these biomarkers may serve as a molecular classifier of Pap smears.

**Electronic supplementary material:**

The online version of this article (doi:10.1186/s13148-016-0263-9) contains supplementary material, which is available to authorized users.

## Background

In 1941, George Papanicolaou published his landmark paper on the use of vaginal smears for the diagnosis of cervical cancer [1]. The road to his discovery and popularization of the Papanicolaou (Pap) smear was a four-decade-long arduous journey starting with experimentation on guinea pigs, then women attending the clinic of Cornell Medical College [2]. Since the development and systemization of cytomorphology for cancer detection by Papanicolaou in 1948, the Pap smear has remained the foundation for cervical cancer screening worldwide. Today, however, low-resource countries continue to lack the infrastructure to sustain a cytology-based screening program, i.e., rapid transport of smears, quality laboratory services, and trained cytopathologists. With ~528,000 new cases worldwide each year, the highest incidence rates of cervical cancer remain in the unscreened, resource-limited regions of Africa, Latin America, Southeast Asia, and the Western Pacific [3].

Since the isolation and cloning of human papillomavirus (HPV)-16 from cervical carcinoma by zur Hausen et al. in 1983, the HPV is now recognized as a necessary cause of invasive cervical cancer with a prevalence of 99 % in global samples [4, 5]. With advancements in molecular diagnostics and automation, primary high-risk HPV (hrHPV) cervical screening and alternative strategies that supplant the resource-demanding cytology-based model, such as visual inspection with acetic acid (VIA), have risen to the forefront. Both screening strategies are now incorporated into the 2014 World Health Organization (WHO) published guidance on cervical cancer [3]. The cobas® hrHPV test, recently approved by the US Food and Drug Administration (FDA) for primary screening, is a qualitative PCR assay that detects HPV types 16 and 18 and/or the other 12 high-risk types [6]. However, this test is limited by the non-specific detection of non-16/18 hrHPV types and non-detection of possibly carcinogenic and not classifiable types as defined by the International Agency for Research on Cancer (IARC) [7, 8]. In contrast, full-spectrum HPV genotyping reveals the genotype and phenotype (carcinogenic potential), which are valuable guides for selecting conservative or ablative therapy in the clinical setting.

Over the last two decades, our understanding of cancer epigenetics has deepened immensely [9]. The body of literature investigating aberrant DNA methylation in cervical carcinoma and its contribution to carcinogenesis via silencing of tumor suppressor genes continue to grow [10–15]. The association between HPV infection and aberrant promoter hypermethylation in host genes appears to be causal. However, quantitative DNA methylation studies of abnormal cervical cytology are sparse, and none has incorporated HPV genotype beyond high-risk types as a predictive marker [16, 17].

To better understand the trilateral relationship between HPV, genomic DNA methylation, and cervical cytopathology, our goal was to use state-of-the-art molecular techniques to screen and profile HPV genotypes and DNA methylation in normal and precancerous Pap smears. The correlation between the predictors (HPV genotype and extent of cellular DNA methylation) and three cytological grades would then be quantitated and explored for its utility as a molecular classifier of cervical cytology.

## Methods

### Subjects and samples

This study was conducted after gaining approval by the Institutional Review Board of the Brooke Army Medical Center (BAMC), Texas. Inclusion criteria were cervical specimens derived from adult women ≥18 years of age undergoing cervical cytology screening. Exclusion criteria were cervical specimens from patients with conditions that may alter genomic methylation, e.g., pregnancy and non-HPV sexually transmitted infections.

Liquid-based cytology collected for clinical testing at the Department of Pathology of BAMC was consecutively procured after completion of analysis for cytological diagnosis. Samples were refrigerated at 4 °C until weekly batch DNA extraction. Demographic data were abstracted from the electronic health record (AHLTA) of the Department of Defense (DoD) and code-linked to each specimen. Three categories of the samples, i.e., negative for intraepithelial lesion or malignancy (NILM), low-grade squamous intraepithelial lesion (LSIL), and high-grade squamous intraepithelial lesion (HSIL), were collected until target accrual numbers were met: NILM (*N* = 100), LSIL (*N* = 100), and HSIL (*N* = 77).

### Cell lines and culture

Five cervical cancer cell lines (SiHa, HeLa Ca Ski, C33-A, and DoTc2) were acquired from the American Type Culture Collection (ATCC) to serve as (+) controls and comparators of methylation. The cell type, tumor site derivation, and HPV status were as follows: SiHa (squamous, primary, HPV16+); HeLa (adenocarcinoma, primary, HPV18+); Ca Ski (squamous, small intestine metastasis, HPV16+/18+); C33-A (epithelial, primary, HPV−); and DoTc2 (epithelial, primary, HPV−). Cells were cultured in flasks for DNA extraction and μ-Slides (Ibidi) for microscopy with appropriate media supplemented with 10 % FBS. EMEM medium (ATCC) was used to grow HeLa, C-33A, and SiHa cells. DMEM and RPMI-1640 media (ATCC) were used to culture DoTc2 and Ca Ski cells, respectively. Cells were grown at 37 °C in a CO_2_ incubator until reaching 80–90 % confluence. For methylation analysis, cellular DNA was extracted for bisulfite conversion and pyrosequencing as described below for cytology samples. For visualization of phenotypic differences, cellular organelles were stained as follows. The mitochondria were stained by incubating cells overnight with fresh media containing 300 nM of MitoTracker® Orange CM-H2TMRos (Life Technologies) followed by washing with fresh media for 15–30 min at 37 °C. Cells were fixed and permeabilized with the FIX & PERM® kit (Life Technologies). Actin and nuclei were stained with respective reagents, ActinGreen™ 488 and NucBlue® (Life Technologies), washed with PBS, and mounted in ProLong® Gold antifade reagent (Life Technologies). Images were acquired by a Leica TCS SP5 II confocal microscope (Leica Microsystems).

### The Cancer Genome Atlas cohort

The cervical cancer cohort of The Cancer Genome Atlas (TCGA) was accessed on 3 October 2014 to acquire DNA methylation data of squamous cell carcinomas (*N* = 231) and adenocarcinomas (*N* = 26). The methylation data (*β* value) generated with the Illumina HumanMethylation450 platform (HM450) in the level 3 format were used to determine promoter methylation levels of adenylate cyclase 8 (*ADCY8*), cadherin 8, type 2 (*CDH8*)*,* O-6-methylguanine-DNA methyltransferase (*MGMT*), and zinc finger protein 582 (*ZNF582*). The matched RNA-SeqVersion 2 expression data [18] were accessed via the cBioPortal [19] to determine the correlation between methylation and expression of the four genes of interest. The few available samples (*N* = 3) with matched (tumor/normal) DNA methylation (accessed on 15 January 2015) were used to compare within and between subject differences.

### Laboratory schema

**Fig. 1:**
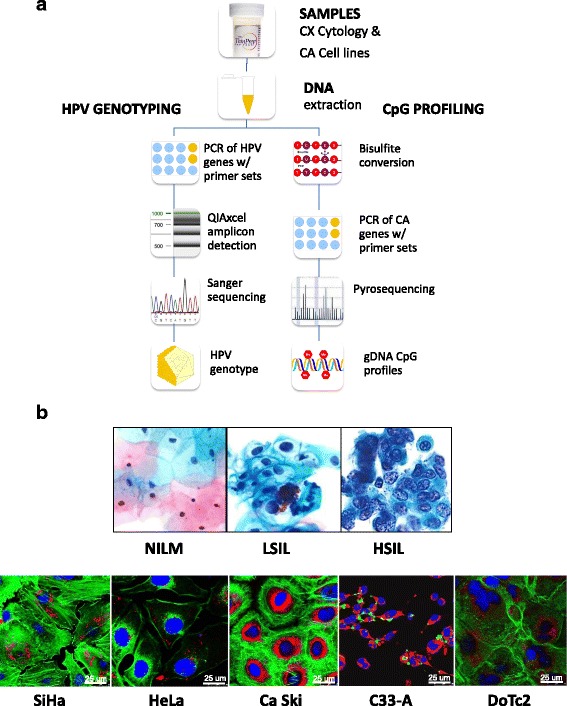
Protocol schema and representative images of cervical cytology and cervical carcinoma cell lines used in the study. **a** Sample collection, DNA extraction, HPV genotyping by Sanger sequencing, and CpG profiling of gene-specific promoters by pyrosequencing. **b** Three categories of liquid-based cervical cytology: negative for intraepithelial lesion or malignancy (NILM), low-grade squamous intraepithelial lesion (LSIL), and high-grade intraepithelial lesion (HSIL), reveal progressive nuclear enlargement, nuclear membrane irregularity, and chromatin coarseness associated with worsening grade. Five cervical carcinoma cell lines: SiHa, HeLa, Ca Ski, C33-A, and DoTc2, with distinct cytomorphologic features, e.g., cell size and shape, nucleus (*blue*), nuclear-to-cytoplasmic ratio, chromatin patterns, actin cytoskeleton (*green*), and mitochondria (*red*). Each cell line was immunofluorescence labeled and imaged by confocal microscopy (×63 objective). *Abbreviations*: *CX* cervical, *CA* cancer, *PCR* polymerase chain reaction, *HSIL* high-grade squamous intraepithelial lesion, *LSIL* low-grade squamous intraepithelial lesion, *NILM* negative for intraepithelial lesion or malignancy

Figure 1a illustrates the laboratory schema. After sample collection, cellular DNA is extracted from cervical cytology or cultured cancer cell lines. The DNA is subjected to HPV DNA amplification, sequencing, and genotyping. For DNA methylation analysis, the genomic DNA undergoes bisulfite conversion and pyrosequencing. The results derived from HPV genotyping and methylation quantification are analyzed for association or correlation with the cytological grade. Figure 1b shows representative images of the three categories of cervical cytology and five immunostained cervical cancer cell lines used in this study. Morphological features and differences are highlighted by the relative size and distribution of organelles, i.e., mitochondria (orange), actin filaments (green), and nuclei (blue).

### HPV DNA amplification

Cervical cytology (10 mL) was centrifuged (4000 rpm × 2 min), and the supernatant was removed. The cell pellet (200–250 μL) was transferred into sample tubes (2 mL) and placed in a QIAcube robotic workstation (Qiagen) for DNA extraction using the QIAamp DNA Mini kit (Qiagen). The purified DNA in 150 μL of the eluent was quantified by spectrophotometry and stored at −20 °C prior to amplification. For HPV DNA amplification, three consensus primer sets: (1) MY09/11, (2) FAP59/64, and (3) GP-E6-3F/GP-E7-5B/GP-E7-6B, were used to amplify two regions of HPV L1 and E6/E7 for genotype identification [20–22]. AmpliTaq Gold 360 Master Mix (Life Technologies) and Qiagen Multiplex PCR Plus kit (Qiagen) were used with the doublet and triplet primer sets, respectively. Briefly, PCRs were performed in a final volume (50 μL) containing template DNA (200 ng), PCR Master Mix (25 μL), forward and reverse primers (1 μM each), and RNAase-free water. The cycling protocols for the three primer sets were as follows: (1) MY09/11: activation (95 °C × 5 min), 40 cycles of three-step cycling (95 °C × 30 s, 57 °C × 90 s, 72 °C × 90 s), final extension (72 °C × 10 min); (2) FAP59/64: activation (95 °C × 5 min), 40 cycles of three-step cycling (94 °C × 60 s, 50 °C × 90 s, 72 °C × 60 s), final extension (72 °C × 10 min); and (3) GP-E6/7: activation (95 °C × 5 min), 45 cycles of three-step cycling (94 °C × 30 s, 55 °C × 90 s, 72 °C × 90 s), final extension (72 °C × 10 min). After amplification, high-resolution capillary gel electrophoresis was used to detect amplicons by the QIAxcel (Qiagen) using the OM500 protocol. Samples with amplicon bands were selected for DNA sequencing.

### HPV DNA sequencing and genotyping

PCR products were purified using the GeneRead Size Selection Kit (Qiagen) on the QIAcube robot. Sanger sequencing of the amplicons (~200 ng DNA/sample) was performed by using sequencing primers MY11, FAP59, and GP-E6-3F (Eurofins Operon). Sequence quality was assessed using the Sequence Scanner 2.0 (appliedbiosystems.com), where a “high-quality” trace score (TS) (average base call quality value) was defined as ≥20 and a QV20+ value (total number of bases in the sequence with TS ≥ 20) as ≥100. Quality sequences were filter selected for entry into the Basic Local Alignment Search Tool (BLAST®) and queried against HPV sequences in GenBank® under virus taxonomy ID# 151340 [23]. The HPV genotype was based on the most homologous and significant result. The proportions of samples in which HPV was detected according to (1) genotype and (2) carcinogenic potential within each cytological category were compared.

### Gene selection and methylation analysis

To confirm and discover new hypermethylated genes in cervical carcinoma, 48 genes were selected for testing (Additional file 1: Table S1). The selection of these genes is addressed in the “Results” section. For the methylation profiling of cervical cytology, the extracted genomic DNA (≥20 ng/μL) was bisulfite-converted using the EZ DNA methylation (Zymo) to convert unmethylated cytosine residues to uracil. The converted DNA in the same cytological category was amassed to generate three pools by using equal amounts (2 μL) from individual samples. Specifically, the first 36, 42, and 18 samples collected from NILM, LSIL, and HSIL categories, respectively, were used for pooled methylation screening [24]. The PCR cycling protocol using the Applied Biosystem polymerase (N12338) was as follows: activation (95 °C × 5 min); 50 cycles of three-step cycling (95 °C × 60 s, 60 °C × 60 s, 72 °C × 60 s); and final extension (72 °C × 7 min). Loci-specific PCR amplification of the pooled DNA (10–20 ng) in technical replicates using Qiagen or PyroMark SW 2.0 designed primers (Additional file 2: Table S2) was followed by pyrosequencing on a PyroMark Q96 MD system (Qiagen). Methylation quantification of each CpG site was performed using the PyroMark CpG 1.0 software. The built-in internal quality control for bisulfite treatment and non-specific background was set to 6.5 %.

The screening criterion used to define hypermethylation at each CpG site was ≥2.0× the methylation level (%) of normal cytology samples. This method is comparable to the selection criteria used by Farkas et al. [25] for *β* values derived from the Illumina HM450 platform. A CpG locus was considered hypermethylated if the Δ*β* value was ≥0.2, and the baseline (normal tissue) was <0.2. Six genes met our screening criteria: *ADCY8, CDH8, ZNF582, MGMT, ALK*, and *NEFL*. The best candidates (first four genes) were selected for further testing of individual samples based on documented association with cervical, oral, and/or endometrial carcinoma [11, 26, 27]. The first 170 consecutively collected cytology samples from the following categories: NILM (*N* = 33), LSIL (*N* = 70), and HSIL (*N* = 67), were subjected to individual locus-specific methylation quantification. Furthermore, the HPV status and methylation levels of these samples were used to construct the multivariable logistic model described below.

### Definitions, variable coding, and logistic modeling

For this study, the classification of HPV carcinogenicity was based on the WHO IARC Working Group Reports [7, 8]. Specifically, HPV types 16, 18, 31, 33, 35, 39, 45, 51, 52, 56, 58, 59, and 68 were deemed carcinogenic (group 1); HPV types 26, 30, 34, 53, 66, 67, 69, 70, 73, 82, 85, and 97 were possibly carcinogenic (group 2B); and HPV types 6, 11, and others were not classifiable or not studied. To compare the prevalence of HPV genotypes grouped by carcinogenicity among the three cytological categories, the HPV genotype found in each sample was coded in an ordinal scale: HPV undetected (0), not classifiable (1), possibly carcinogenic (2), and carcinogenic (3). Cytology was also coded on an ordinal scale, NILM (0), LSIL (1), and HSIL (2), to determine the correlation between HPV carcinogenicity and cytological grade.

Multivariable logistic regression [28] was performed to investigate the association between the methylation level of each CpG locus of a particular gene (*ADCY8, CDH8*, and *ZNF582*) and a binarized cytological outcome of interest. Outcome model 1 aimed to distinguish normal from abnormal cytology (NILM vs. LSIL/HSIL), whereas model 2 distinguished between non-high and high-grade cytology (NILM/LSIL vs. HSIL). The model equation is as follows:

Logistic model: Probability of outcome = P(Y = 1) = 1/(1 + *e*^∧^(−(b0 + b1X1 + ⋯ + b*i*X*i*)))

Multiple explanatory variables: $$ \begin{array}{cc}\hfill \mathrm{X}1,\dots, \mathrm{X}i\hfill & \hfill \left( Xi=\mathrm{Gene}\kern0.2em \mathrm{Xand}\kern0.5em \mathrm{C}\mathrm{p}\mathrm{G}\hbox{-} \mathrm{position}\kern0.5em i\kern0.5em \mathrm{methylation}\kern0.5em \mathrm{level}\kern0.5em \left(\%\right)\right)\hfill \end{array} $$

Model 1 outcome (Y) coding: NILM (0), LSIL/HSIL (1)

Model 2 outcome (Y) coding: NILM/LSIL (0), HSIL (1)

The covariates (CpG position selected from each gene) that had the highest association with the response variable (lowest *P* value) were selected for cut-point (binarization) determination. The cut-points were chosen at the point of maximum accuracy (∑ sensitivity + specificity). The new binarized methylation variables of these CpG sites, along with HPV carcinogenic status, were entered in a second multivariable logistic regression analysis to select the explanatory variables most predictive of the cytological outcome. The second model equation is as follows:

Logistic model: Probability of outcome = P(Y = 1) = 1/(1 + e^∧^(−(b0 + b1X1 + ⋯ + b4X4)))

Multiple explanatory variables: X1, …, X4

X1 = HPV carcinogenicity (coded as ordinal data as described in text)

X2 = ADCY8 CpG-position *i* methylation (0, 1)

X3 = CDH8 CpG-position *i* methylation (0, 1)

X4 = ZNF582 CpG-position *i* methylation (0, 1)

Model 1 outcome (Y) coding: NILM (0), LSIL/HSIL (1)

Model 2 outcome (Y) coding: NILM/LSIL (0), HSIL (1)

For the final regression models, post-estimation receiver operating characteristic (ROC) curves were constructed and predictions at specified values were computed. After estimating the classification threshold or “cut-point” for each model by using the maximum sum of sensitivity and specificity, diagnostic performance characteristics were determined. The discriminatory performance between multivariable and univariable (HPV carcinogenicity only) models was compared using respective areas under the ROC curve. Pairwise comparisons of predicted probabilities between models were performed with the chi-square test.

### Statistical analysis

This study was designed to have an 80 % power to detect a 20 % difference in DNA methylation (%) between normal and abnormal cytology. From the literature, locus-specific promoter methylation levels (%) for NILM, LSIL/HSIL, and cervical cancer have ranged from 0 to 5 %, 15 to 30 %, and 30 to 60 %, respectively [13, 15, 29]. To detect a 20 % difference in methylation levels using a one-sided test set at *α* = 0.05 and *β* = 0.20 with an allocation ratio of 2 (N2/N1), a minimum accrual target of N2 = 62 and N1 = 31 per group was required. The quota sampling strategy assured adequate representation from each cytological grade. Furthermore, additional samples were collected to account for potential sample inadequacy and laboratory errors.

Data were summarized using means (95 % CI), medians (IQR), and proportions. For hypothesis testing, Wilcoxon rank sum and Kruskal-Wallis tests were used for non-parametric, numerical, or ordinal data. Categorical data were compared using the chi-square test. Correlation between ordinal variables was determined by Spearman’s rho. *P* values <0.05 were considered statistically significant.

For TCGA methylation analysis, the pyrosequencing CpG assay for each gene was translated into the Illumina assay by selecting the nearest CpG loci on the HM450K array. Methylation data (*β* value, defined as the ratio of methylated signal over the total signal (methylated + unmethylated)) [25] were used to determine promoter methylation levels of *ADCY8, CDH8, MGMT*, and *ZNF582*. The median methylation values per locus were stratified by the observation group, i.e., tumor stages and histologic category (normal/tumor), and tested for differences by non-parametric methods. All subsequent analyses compared median methylation values across all CpGs per gene as the single sample summary measure. The correlation between methylation (*β* value) and RNA-SeqV2 expression data (upper quartile of normalized RSEM count estimates) [18] was determined by Spearman’s rho. Statistical analyses were performed using STATA/IC 13.0 (StataCorp LP).

## Results

### HPV carcinogenic genotypes are correlated with HSIL

Clinical and cytological characteristics are summarized in Table 1. Residual cytology samples (*N* = 400) were collected between January 2013 and 2014. Of all samples, 31 % (*N* = 123) were excluded because of low quantity, low quality, or sample excess, as described in Table 1. For samples that met inclusion criteria (*N* = 277), the corresponding subjects were composed predominantly of Caucasians (45 %) with a median age of 28 years (IQR, 24–35). The cytological specimens were stratified proportionately among the three grades: NILM 100/277 (36 %), LSIL 100/277 (36 %), and HSIL 77/277 (28 %). The median concentration of the extracted DNA among the three cytological categories (range, 46.3–51.8 ng/μL) was statistically equivalent (Kruskal-Wallis test, *p* = 0.519) (Table 1).

**Table 1:** Clinical and cytological characteristics of the study population

Characteristics	Number	Percentage^g^
Clinical		
Age^a^		
Median (IQR)	28	(24–35)
Range (year)	19–67	
Race/ethnicity^a^		
Asian [NILM, LSIL, HSIL]	9 [5, 3, 1]	(3)
Black [NILM, LSIL, HSIL]	40 [14, 15, 11]	(14)
White [NILM, LSIL, HSIL]	124 [48, 40, 36]	(45)
Other [NILM, LSIL, HSIL]	65 [21, 28, 16]	(24)
Unknown [NILM, LSIL, HSIL]	39 [12, 14, 13]	(14)
Cytological^b^		
Total LBC samples collected	400	(100)
LBC samples excluded^c^	123	(31)
NILM	33	
LSIL	85	
HSIL	5	
LBC samples included	277	(69)
NILM	100	
LSIL	100	
HSIL	77	
Source^d^		
Cervical	276	(99.6)
Vaginal	1	(0.4)
Diagnostic category^d^		
Normal	100	(36)
Abnormal	177	(64)
Cellular DNA concentration^d,e,f^		
NILM	100	
Median (ng/μL) (IQR)	48.1	(37.1–74.3)
Range (ng/μL)	20.8–181.5	
LSIL	100	
Median (ng/μL) (IQR)	46.3	(35.6–63.1)
Range (ng/μL)	20.5–182.8	
HSIL	77	
Median (ng/μL) (IQR)	51.8	(34.9–71.3)
Range (ng/μL)	11.5–154.0	

*IQR* interquartile range, *LBC* liquid-based cytology, *LSIL* low-grade squamous intraepithelial lesion, *HSIL* high-grade squamous intraepithelial lesion, *NILM* negative for intraepithelial lesion and malignancy

^a^Age and race/ethnicity of subjects (*N* = 277) are based on the demographic data of included samples. The number of samples by race/ethnicity and cytological diagnosis are placed in square brackets. The distribution of the five categories of race/ethnicity (inclusive of *Other* and *Unknown*) among the three cytological grades were not significantly different (*χ* ^2^, *p =* 0.777)

^b^Cytopathology results are ascribed to the specimens collected on the day of the study enrollment

^c^Exclusion criteria included: low cell pellet volume (<200 μL); low cellular DNA concentration (<20 ng/μL); low nucleic acid purity (spectrophotometry absorbance ratio 260/230 nm <0.7); excess samples (>100) (see text for details)

^d^Data based on included samples (*N* = 277)

^e^Concentration of total cellular DNA per sample after manual or semi-automated DNA extraction

^f^Comparison of DNA concentrations between NILM, LSIL, and HSIL samples were not significantly different (*p =* 0.519) by Kruskal-Wallis test

^g^Values are *N* (%) unless otherwise denoted

**Fig. 2:**
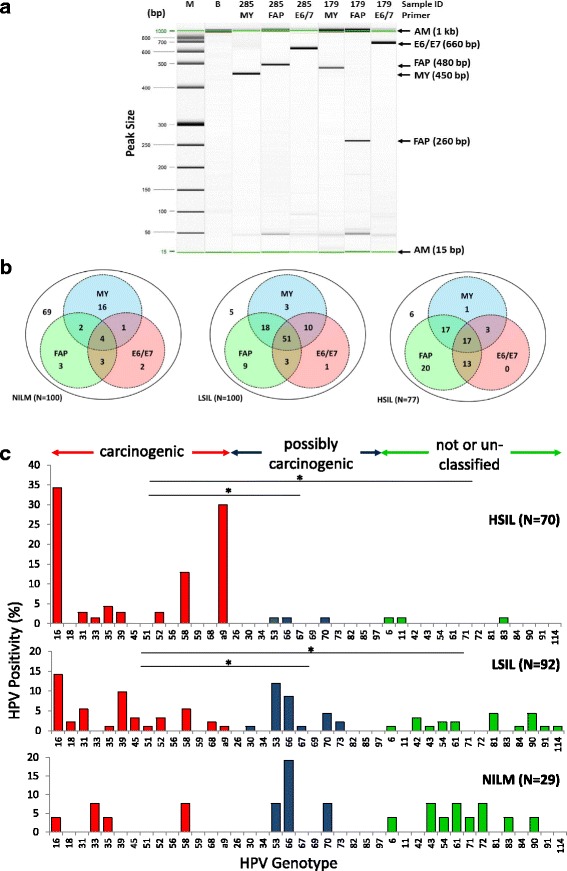
PCR amplification of HPV DNA by three consensus primer sets and HPV genotyping by amplicon sequencing. **a** Representative gel image of PCR amplicon detection by high-resolution capillary gel electrophoresis. Representative samples #285 (LSIL) and #179 (HSIL) reveal MY09/11, FAP59/64, and GP-E6/E7 F/B amplicons with expected yield of ~450-, 480- (or 260-bp fragment), and 660-bp fragments, respectively. **b** Parallel PCR testing for HPV by three primer sets. Venn diagrams show intersecting and complementary sets of cytological samples (*N*) detected of HPV DNA by MY-, FAP-, and E6/E7 primer sets according to cytological diagnoses, i.e., NILM, LSIL, and HSIL. The net positivity of simultaneous testing for HPV (*union of the circles*) in NILM, LSIL, and HSIL are 31/100 (31 %), 95/100 (95 %), and 71/77 (92 %), respectively. **c** HPV genotype distribution of 191 cytology samples with PCR-detected HPV DNA according to cytological diagnoses: NILM, LSIL, and HSIL. The increase in carcinogenic HPV genotypes was coincident with cytological grade (Spearman’s *ρ* = 0.658, *p* < 0.001). Samples positive for the 260-bp fragment that aligned closest to HPV-58 were assigned as “alpha-9” species because of the non-specific short sequence length. *p < 0.05 by the chi-square test. *Abbreviations*: *AM* alignment marker, *B* buffer, *bp* base pair, *HSIL* high-grade squamous intraepithelial lesion, *IARC* International Agency for Research on Cancer, *LSIL* low-grade squamous intraepithelial lesion, *M* molecular weight ladder, *NILM* negative for intraepithelial lesion or malignancy

To optimize HPV DNA detection, three primer sets targeting three distinct regions of the HPV genome were used. PCR amplification using primers MY09/11, FAP59/64, and E6-E7 F/B yielded the expected 450-, 480-, and 660-bp fragments upon capillary gel electrophoresis (Fig. 2a). An unexpected short amplicon (260 bp) derived from amplification with the FAP primers was observed at higher frequency in HSIL samples. DNA sequencing and nucleotide BLAST mapped the 260-bp sequence nearest to the HPV-58L1 segment (nucleotide range 6041 to 6253) belonging to the alpha-9 species but non-specific for genotype identification. Partial loss of the HPV L1 gene was presumed because of virus-to-host genome integration frequently found in HSIL [30].

Gel electrophoresis positivity for HPV DNA after PCR of each sample by the three primer sets is summarized by intersecting and complementary sets within Venn diagrams in Fig. 2b. The combined net positive rates of HPV DNA detection for NILM 31/100 (31 %), LSIL 95/100 (95 %), and HSIL 71/77 (92 %) are represented by the union of three sets within each Venn diagram (Fig. 2b). Of the PCR-positive samples that were sequenced, 191 samples were genotyped by BLAST [23].

The prevalence of HPV genotypes found in three grades of cytology is shown in Fig. 2c. The genotype spectrum spanned the continuum of IARC-defined carcinogenic potentials. As expected, there was a higher frequency of HPV16 genotypes detected in low- and high-grade cytology. Notably, the proportion of carcinogenic HPV types positively correlated with cytological grade: NILM (23 %), LSIL (49 %), and HSIL (91 %). Furthermore, LSIL and HSIL samples had a significantly greater proportion of carcinogenic than possibly carcinogenic and not classifiable HPV genotypes (chi-square, *p* < 0.05), whereas the distribution did not vary among NILM. Finally, a high frequency of HPV-58 was noted in HSIL samples.

### Promoter hypermethylation of *ADCY8, CDH8,* and *ZNF582* are correlated with HSIL

**Fig. 3:**
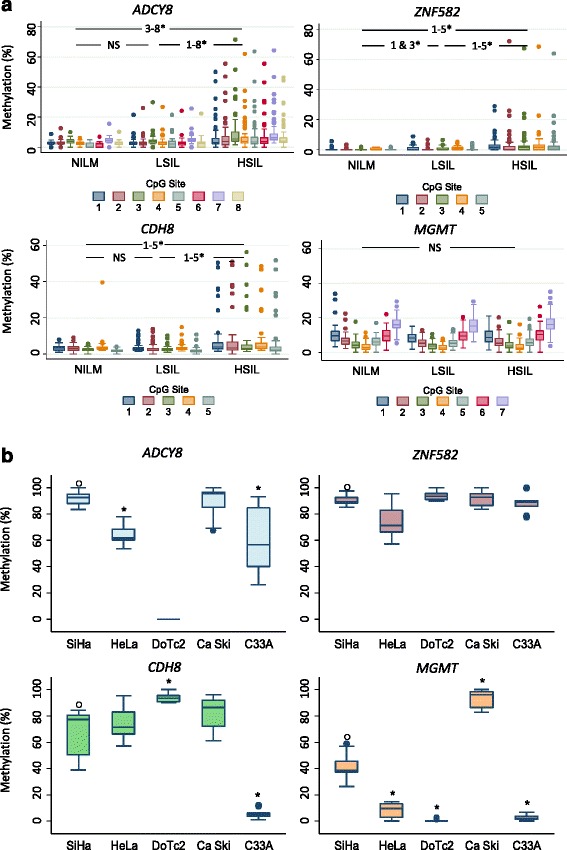
Promoter methylation differences in cervical cytology and cervical carcinoma cell lines. **a** Methylation (%) of total genomic DNA in three grades of cervical cytology, i.e., NILM (*N* = 33), LSIL (*N* = 70), and HSIL (*N* = 67), was compared by CpG positions among four genes (*ADCY8*, *CDH8*, *ZNF582*, and *MGMT*). Pairwise comparisons of methylation for each CpG position between cytological grades (NILM vs. LSIL, LSIL vs. HSIL, and NILM vs. HSIL) revealed significantly higher levels for HSIL vs. LSIL and LSIL vs. NILM at multiple positions for *ADCY8*, *CDH8*, and *ZNF582*. For *MGMT*, methylation levels were not significantly different among cytological grades. Methylation levels for each CpG position increased concurrently with cytological grade for *ADCY8*, *CDH8*, and *ZNF582* by Spearman’s *ρ (p* < 0.001*).* *p < 0.05 by the Wilcoxon rank-sum test. **b** SiHa, HeLa, and Ca Ski cell lines with genome-integrated HPV demonstrated promoter hypermethylation of *ADCY8*, *CDH8*, and *ZNF582* genes. For HPV-negative cell lines, DoTc2 and C33-A revealed an inconsistent pattern of hypermethylation in the studied genes. Using SiHa methylation (%) as a reference (o), cell lines with significantly different levels are indicated by an *asterisk*. *p < 0.05 by the Wilcoxon rank-sum test. *NS* not statistically significant. Cell lines were analyzed for CpG methylation in duplicate collections

The panel of genes (Additional file 1: Table S1) selected for promoter methylation screening was composed of genes previously reported to be hypermethylated in cervical carcinoma and other malignancies, e.g., brain, oral, breast, lung, hepatocellular, colorectal, and endometrial. Many of these genes are known to participate in the six biological capability hallmarks of cancer, making them plausible factors in cervical carcinogenesis [31]. The quantitative methylation results of four candidate genes selected for pyrosequencing stratified by Pap grade and CpG position are presented in Fig. 3a. The results indicate a positive correlation between Pap grade and promoter methylation of *ADCY8, CDH8*, and *ZNF582* (Spearman’s rank, *p* < 0.05) but not *MGMT*. Pairwise comparison of methylation at each CpG locus between Pap grades revealed higher levels in HSIL than LSIL and NILM with a few exceptions (Fig. 3a). The differences between LSIL and NILM were only significant for *ZNF582* CpG loci 1 and 3 (*) (Fig. 3a). Interestingly, for *MGMT*, methylation values did not vary across Pap grades and CpG positions.

### Promoter hypermethylation of *ADCY8, CDH8,* and *ZNF582* is validated in cervical cancer cell lines and TCGA cohort

Promoter methylation of four candidate genes was quantified in five cervical cancer cell lines. The median methylation across all CpG sites for each gene stratified by cell line is presented in Fig. 3b. In general, hypermethylation of *ADCY8, CDH8*, and *ZNF582* was noted in all cell lines except C33A and DoTc2 (which failed the *ADCY8* assay). For comparison between cell lines, the methylation levels of all four genes in SiHa (ranging from ~38 % in *MGMT* to 93 % in *ADCY8*) were used as the referent. Although some significant differences in DNA methylation levels were detected, e.g., decreased methylation of *ADCY8* in HeLa/C33A cells and *CDH8* in C33A cells (Fig. 3b), the HPV-positive cell lines consistently exhibited high methylation levels (>50 %). For *MGMT*, methylation levels among the cell lines were inhomogeneous and polarized (Fig. 3b).

**Fig. 4:**
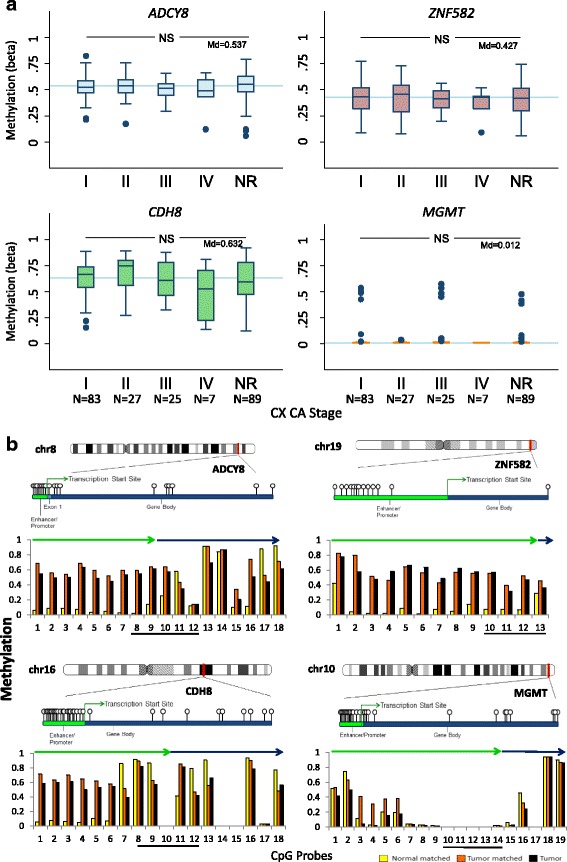
Promoter methylation of *ADCY8*, *CDH8*, *ZNF582*, and *MGMT* in the TCGA cervical cancer cohort. **a** Box plots of CpG methylation (*β* value) according to FIGO stage for 231 patient samples with squamous cell carcinoma. Gene-specific median methylation values for all FIGO stages are specified (Md) and indicated by the *blue reference lines. NS* not statistically significant, Kruskal-Wallis *P >* 0.05. *NR* stage not reported. **b** Differential CpG methylation (*β* value) ante- and post-transcription start site for 257 cervical carcinomas (squamous, *N* = 231; adenocarcinoma, *N* = 26) and 3 tumor/matched normal samples. The four panels display the chromosomal positions of *ADCY8*, *CDH8*, *ZNF582*, and *MGMT* (*red line*) with an expanded area showing the CpG probes on the Illumina HumanMethylation 450 K microarray (gene ball-and-stick diagrams). The *bar graphs* present the median DNA methylation (*β* value) of 257 tumors (*black*) and 3 matched tumor (*orange*)/normal (*yellow*) samples across the ordered CpG probes. The promoter methylation levels were notably higher (~×10) for tumor (median *β* ~0.6) than the normal samples (median *β* ~0.06) for *ADCY8*, *CDH8*, and *ZNF582*. The enhancer/promoter and gene body regions are indicated by the *green* and *blue arrows*, respectively. The CpG regions selected for bisulfite pyrosequencing of cytology samples are denoted by the underscored CpG probes. The chromosome coordinates for the CpG probes along the *X-axis* are as follows: *ADCY8* (chr8: 132,053,823-131,896,788), *CDH8* (chr16: 62,070,072-61,871,849), *ZNF582* (chr19: 56,905,383-56,901,457), and *MGMT* (chr10: 131,264,840-131,304,833). [Chromosome ideograms adapted from NCBI Map Viewer (www.ncbi.nlm.nih.gov/genome/guide/human)]

TCGA data for the cervical cancer cohort (*N* = 231) revealed distinct hypermethylation patterns among *ADCY8, ZNF582*, and *CDH8* (Fig. 4a) for reported and non-reported clinical stages (median *β* value range, 0.427–0.632). For MGMT, the methylation was consistently low with a median *β* value of 0.012 across all stages. Moreover, methylation levels were not distinguishable between stages for the four genes (Kruskal-Wallis, *p* > 0.05). Association analysis between methylation and matched RNA-Seq expression data revealed modest anti-correlation for *ZNF582* (Spearman’s *ρ* = −0.2349, *p* < 0.05) and *MGMT* (Spearman’s *ρ* = −0.1660, *p* < 0.05) but not for *ADCY8* and *CDH8* (Additional file 3: Figure S1).

TCGA data for the three available tumor/normal matched pairs of cervical tissues were examined for within and between subject promoter methylation differences. Because of the small sample size, formal statistical analysis was not performed. However, increased median methylation (~10×) of *ADCY8, CDH8*, and *ZNF582*, but not *MGMT*, was noted in the tumor cohort (*N* = 257) compared with the three normal samples (Fig. 4b). Notably, the methylation levels for adenocarcinomas (*N* = 26) were comparable to those of squamous carcinomas; hence, these samples were included in the tumor cohort.

### HPV genotype and promoter hypermethylation of *ADCY8, CDH8,* and *ZNF582* as a predictor of HSIL

**Fig. 5:**
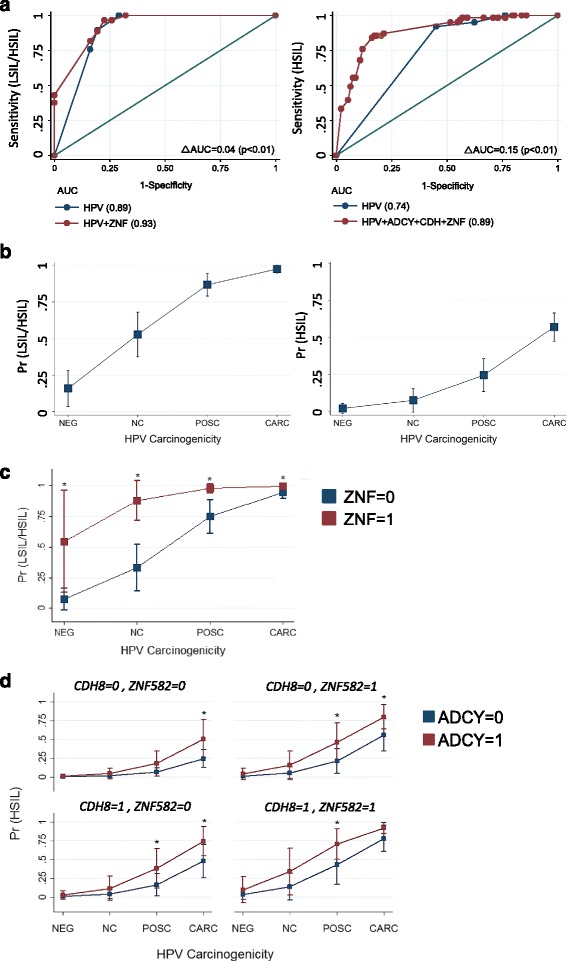
Regression models and predicted probability plots for cytological grades. **a** Receiver operating characteristic curve analysis using cut-points derived from univariate ROC analysis of gene-specific methylation levels. Multivariable modeling revealed the best predictor to differentiate between NILM and LSIL/HSIL was HPV carcinogenicity and *ZNF582*_7th CpG position binarized as follows: <1.1 (0), ≥1.1 (1) (ROC AUC = 0.93). For differentiating between NILM/LSIL and HSIL cytology, the best multivariate predictor was the combination of HPV carcinogenicity, *ADCY8*_7th CpG-position, *CDH8*_3rd CpG-position, and *ZNF582*_3rd CpG-position (ROC AUC = 0.89); the binarized methylation values (%) used for the respective three genes were as follows: <5.8 (0), ≥5.8; <3.0 (0), ≥3.0 (1); and <1.1(0), ≥1.1(1). **b** Predicted probability plot of binarized cytology grades (NILM vs. LSIL/HSIL and NILM/LSIL vs. HSIL) using HPV carcinogenicity as the single predictor variable. **c** Comparison of predicted probabilities for abnormal cytology (NILM vs. LSIL/HSIL) by HPV carcinogenicity and binarized *ZNF582* methylation level coded as <1.1 (0) or ≥1.1 (1). **d** Comparison of predicted probabilities for HSIL (NILM/LSIL vs. HSIL) permuted by binarized methylation values of *ADCY8, CDH8,* and *ZNF582* at the CpG positions noted above. The four panels illustrate the escalating probability for HSIL coincident with the increasing number of methylated genes. **a–d** The number of cytology samples grouped by HPV carcinogenic potential among a total of 170 samples were as follows: negative (*N* = 29), not classifiable (*N* = 18), possibly carcinogenic (*N* = 18), and carcinogenic (*N* = 105). *p < 0.05 by the chi-square test and delta method for pairwise comparison of margins. *ROC* Receiver operating characteristic, *AUC* area under the curve

The logistic regression analysis and ROC curves for the univariable and multivariable logit models for cytological outcomes are presented in Additional file 4: Table S3, Additional file 5: Table S4, and Fig. 5a, respectively. The HPV carcinogenic potential (carcinogenic, possibly carcinogenic, not classifiable, and negative) among the three cytological categories was distributed, respectively: HSIL (91, 3, 3, 3 %); LSIL (54, 21, 17, 7 %), and NILM (18, 3, 12, 67 %). For model 1, the best predictors were HPV carcinogenicity and *ZNF582*_CpG-position 3, with an area under the ROC of 0.93. For model 2, the best predictors were HPV carcinogenicity and *ADCY8*_CpG-position 7, *CDH8*_CpG-position 3, and *ZNF582*_ CpG-position 3, with an area under the ROC of 0.89. The discriminatory performance of both multivariable models inclusive of methylation markers was better than that of the univariate predictor (HPV carcinogenicity) model by comparing areas under the ROC (chi-square, *p* < 0.05).

The predicted probabilities at representative values over the range of predictor variables are presented as margins plots (Fig. 5b–d). Figure 5b, c illustrates the segregating effect of *ZNF582* over HPV carcinogenicity alone as a predictor of abnormal Pap smear (LSIL/HSIL). More importantly, HPV negativity in conjunction with low *ZNF582* methylation was highly indicative of a normal Pap with a negative predictive value (NPV) of 100 % (Fig. 5c). The predicted probabilities or margins for all possible combinations (*N* = 8) of predictor variables in model 1 are provided in Additional file 6: Table S5. For model 2, the cumulative effects of *ADCY8, CDH8*, and *ZNF582* promoter methylation over HPV carcinogenicity alone as a predictor of HSIL were significant (Fig. 5b, d). The probability of HSIL increased incrementally as the number of methylated genes increased from 0 to 3 (Fig. 5d, four-panel chart). The predicted probabilities for all possible combinations (*N* = 32) of predictor variables in model 2 are tabulated in Additional file 7: Table S6.

The diagnostic performance characteristics of models 1 and 2 are presented in Additional file 8: Table S7 and Additional file 9: Table S8. For clinical performance, the sensitivity of HPV + *ZNF582* was higher (100 %) than that of HPV (90 %) in detecting abnormal (LSIL/HSIL) cytology. The positive predictive values (PPVs) were comparable at 93–95 %, suggesting that for patients with a positive assay, almost all have abnormal cytology. In contrast, for patients with a negative assay, the chance of finding no disease (NPV) was 100 % for HPV + *ZNF582* vs. 66 % for HPV, suggesting that HPV + *ZNF582* is a better screening test. For model 2, the PPV was greater for the HPV + three-methylation marker (81 %) vs. HPV (58 %), suggesting that in patients with a positive multi-marker test, almost 80 % will have HSIL. Furthermore, the false-positive rate is lower for the HPV + three-methylation marker (19 %) than for HPV (42 %). Essentially, the results of the two models indicate that (1) HPV + *ZNF582* is a better predictor of NILM and (2) HPV + three-methylation marker is a better predictor of HSIL than HPV alone.

## Discussion

This study aimed to determine the association between HPV genotypes and cellular epigenetic modifications in three grades of cervical cytology. Indeed, our study found positive correlations between HPV carcinogenicity; aberrant DNA methylation in the promoters of *ADCY8*, *CDH8*, and *ZNF582*; and cytological grade. Our previous experience had shown that parallel PCR testing with multiple primer sets optimizes the sensitivity and breadth of HPV detection; thus, this methodology was used herein [23]. The HPV positivity rate detected in normal cytology was 31 %, which increased precipitously to >90 % in LSIL and HSIL samples. Compared with a meta-analysis of worldwide HPV prevalence in normal cytology, our statistic was ~10 % higher [32]. Our extended breadth of detection may be accounted for by the triple-primer PCR approach versus the single-primer PCR and hybrid capture 2 used in the majority of the studies cited [32]. PCR/sequencing was used to determine the dominant HPV genotype within each sample. However, a drawback of direct sequencing is the indecipherability of non-dominant sequences in mixed infections. Although the rate of mixed HPV infections is unknown for our samples, it is noteworthy to recognize the high prevalence of multiple HPV types in NILM, LSIL, and HSIL cytology which may reach 37, 76, and 66 %, respectively, in HPV-positive samples [33, 34]. Furthermore, HPV-58 accounted for a significant proportion (13 %) of carcinogenic HPV in the HSIL category. The high prevalence of HPV-58 may be explained by our population. According to the 2010 Bureau of the Census, 63 % of the population of San Antonio, Texas, is of Hispanic/Latino origin. Ethnogeographical predilection of HPV-58 has been observed in certain Latin American countries, including Southeastern Mexico, Brazil, and Costa Rica [35]. The race/ethnicity of our population derived from electronic medical records indicated that 38 % were categorized as “Other” or “Unknown.” Based on our clinic population, we surmise that “Other” was a person of Hispanic/Latino origin.

The proportion of carcinogenic HPV genotypes found in the samples after genotyping was highest among the HSIL group. Cellular genomic analyses revealed a significant increase in the promoter methylation of *ADCY8*, *CDH8*, and *ZNF582* concomitant with worsening cytological grade. Conjointly, HPV carcinogenicity and the binarized methylation levels of the three genes were significant predictors of cytological outcome in a multivariable model. Specifically, HPV and *ZNF582* demonstrated high discriminatory performance as a screening test to differentiate normal (NILM) from abnormal cytology (LSIL/HSIL) with a NPV of 100 %. In contrast, the lower NPV (66 %) for HPV alone in detecting abnormal cytology may be explained by the elevated false-negative rates of HPV DNA detection in LSIL/HSIL. PCR non-detection may be attributed to several variables, e.g., insufficient DNA template quality or quantity, primer-target mismatch, and loss of HPV viral sequences except for E6 and E7 upon integration into the cellular genome, notably in HSIL and invasive disease [23, 30]. In fact, a recent study by Blatt and colleagues revealed a significant HPV non-detection rate in women with abnormal cytology (14.5 %) and invasive cancer (18.6 %) [36]. For abnormal cytology, HPV and *ADCY8*, *CDH8*, and *ZNF582* differentiated the <HSIL from HSIL samples with a PPV of 81 %. In terms of clinical utility, the addition of quantitative methylation markers to the probabilistic model significantly improved the diagnostic accuracy of HPV carcinogenicity as a single predictor of cytological outcome.

Promoter hypermethylation of *ADCY8, CDH8,* and *ZNF582* was corroborated in five cervical cancer cell lines with two exceptions. C33A cells exhibited low *CDH8* methylation levels and DoTc2 failed the *ADCY8* assay, presumably because of low levels as well. Both C33A and DoTc2 cells are HPV-negative, which may explain the hypomethylation as previously demonstrated in HPV+/HPV− head and neck squamous cell carcinoma (HNSCC) cell lines and tumors [26]. TCGA dataset confirmed gene-specific hypermethylation in cervical tumors. Promoter methylation of *ADCY8, CDH8,* and *ZNF582* was markedly elevated across all four stages of cervical carcinoma. The lack of variability between stages suggested that these epimutations occurred early in the neoplastic process. Whether these alterations are tumor “drivers” or “passengers” is unknown. Nonetheless, they serve as informative host biomarkers for epithelial dysplasia/neoplasia. Moreover, within-subject analysis of matched tumor and normal tissues verified differential promoter methylation for *ADCY8*, *CDH8,* and *ZNF582.* It is worth mentioning that when the current study was initiated, there were only 119 cases (year 2011 (*N* = 66), year 2012 (*N* = 53)) with 1 matched (tumor/normal) sample in TCGA. Furthermore, the targeted CpG loci between pyrosequencing and HM450 methylation assays may not be identical and may thus render different results. Different CpG positions, even in close proximity, within the same CpG-island may exhibit dissimilar methylation levels [37].

The gene products of *ZNF582, CDH8,* and *ADCY8* have unique cellular functions that may be repressed via epigenetic modifications and participate in the neoplastic process. First, *ZNF582* located on chromosome 19 encodes a nuclear protein belonging to the Cys2His2-(C2H2) zinc finger protein family with a conserved Kruppel-associated box (KRAB) domain [38]. The exact function of the *ZNF582* protein is unknown; however, KRAB-ZFPs, in general, are transcriptional repressors that bind to the gene promoter regions via their sequence-specific DNA binding motifs. Some KRAB-ZFPs are known to regulate apoptosis and act as tumor suppressors; thus, inactivation may be involved in tumorigenesis [39]. Previous studies have shown that *ZNF582* is frequently methylated in invasive squamous and adenocarcinoma of the cervix, as well as preinvasive disease [11, 16, 40]. The *CDH8* gene on chromosome 16 encodes cadherin, which is a cell membrane-spanning protein that mediates cell-cell adhesion and recognition [41]. A recent study of HNSCC showed that ten genes of the cadherin superfamily including *CDH8* were hypermethylated in HPV+ HNSCC [26]. Additionally, the HPV E6 gene was identified as the effector gene causing the hypermethylation signature. Silencing of the cadherin superfamily genes has been implicated in many cancers, with attendant hallmarks such as epithelial-mesenchymal transition (EMT) involved in invasion and metastasis [26, 42]. The third gene, *ADCY8* on chromosome 8, encodes a membrane-bound enzyme that catalyzes the formation of cyclic AMP from ATP [43]. This gene is expressed primarily in the brain, and its exact function is unclear. Recent studies have demonstrated its role in brain glioma formation and association with endometrial cancer. Warrington et al. [44] elegantly demonstrated how suppression of cAMP induces gliomatosis and restitution-inhibited glioma growth in a neurofibromatosis-1 mouse model. *ADCY8* hypermethylation and altered expression have also been observed in endometrial cancer [27, 45]. In summary, our findings of hypermethylation in these particular genes are consistent with the existing literature pertaining to methylation, biological function, and plausible roles in carcinogenesis.

The strength of this study lies in the methodologies used for HPV detection and methylation quantification. HPV detection by parallel PCR/sequencing offers the greatest sensitivity and breadth of HPV detection. This method unleashes the constrained spectrum of HPV genotypes detected by commercial tests to obviate measurement bias. Furthermore, allocating the HPV genotypes by IARC-defined carcinogenicity enumerates oncogenic potential to allow for predictive modeling. In contradistinction, commercially available HPV tests only detect *carcinogenic* and not *possibly* or *not classifiable* HPV genotypes. Such dichotomized classification, i.e., high-risk positive or negative HPV, has a significant false-negative rate because of the non-detection of “low-risk” HPV, which may pose a clinical risk. Regarding quantitative DNA methylation, CpG analysis by pyrosequencing was chosen for its accuracy and high quantitative resolution. This method may also be easily translated into a clinically applicable test, i.e., real-time PCR with high-resolution melt analysis [46]. Essentially, the combination of biomarkers has emerged as a refinement of our current one-dimensional clinical diagnostics, i.e., Pap or hrHPV, that serve as markers for detecting and quantifying oncogenic potential. Because this study was conducted as a biomarker discovery project, the ~300 samples used were considered the “training set” for predictive modeling. To overcome this limitation, cross-validation using another larger cohort is underway to predict the fit of our model. Another noteworthy limitation is the use of cytology instead of histology as the outcome of interest for our predictive models. Cytopathology was used as the surrogate marker for the disease due to the total or frequent absence of tissue biopsies for women with NILM and LSIL cytology, respectively. For the cytology samples used for model building, histopathology was available for 29/70 (41 %) LSIL and 52/67 (78 %) HSIL samples with substantial cytohistological agreement rates of 66 and 73 % to CIN I and CIN II–III, respectively. Therefore, cytological diagnosis was deemed a practical and valid outcome measure for model construction. Finally, atypical squamous and glandular cells of undetermined significance (ASC-US and AGUS) cytological categories were not studied. The overall frequency of HPV+/ASC-US (1.1 %) and HPV+/AGUS (0.05 %) is low; however, the 5-year risk of histologic HSIL and cancer is significant, i.e., 18 and 45 %, respectively [47]. To fill this knowledge gap, we plan to investigate uncommon cytological categories to further our understanding of viral ecology and associated epigenetic alterations.

## Conclusions

In conclusion, the results of this study showed that different grades of cervical cytology possess different molecular signatures, which may be translated into a multi-targeted “molecular Pap” for clinical use. With the rapid evolution of molecular technologies, it is foreseeable that cervical cancer screening may become a fully automated, computerized, molecular diagnostic test that may circumvent economic hardships and the non-existence of infrastructures for cytology-based screening programs in developing countries.

## Additional files

Additional file 1: Table S1. Genes screened for promoter methylation in cervical cytology samples [48–52]. (PDF 132 kb) Additional file 2: Table S2. List of pyrosequencing assays. (PDF 186 kb) Additional file 3: Figure S1. DNA methylation and gene expression of *ADCY8, CDH8, ZNF582*, and *MGMT* in the TCGA cervical cancer cohort. (PDF 106 kb) Additional file 4: Table S3. Logistic regression analysis of HPV and HPV + *ZNF582* for predicting abnormal (LSIL/HSIL) cytology. (PDF 151 kb) Additional file 5: Table S4. Logistic regression analysis of HPV and HPV + three-gene methylation markers for predicting for HSIL cytology. (PDF 154 kb) Additional file 6: Table S5. Predictive margins for abnormal (LSIL/HSIL) cytology based on HPV or HPV + *ZNF582*. (PDF 162 kb) Additional file 7: Table S6. Predictive margins for HSIL cytology based on HPV or HPV + three-gene methylation markers. (PDF 196 kb) Additional file 8: Table S7. Diagnostic performance of HPV vs. HPV + *ZNF582* for abnormal (LSIL/HSIL) cytology. (PDF 152 kb) Additional file 9: Table S8. Diagnostic performance of HPV vs. HPV + three-gene methylation markers for HSIL cytology. (PDF 152 kb)

## Abbreviations

*ADCY8*: Adenylate cyclase 8
*CDH8*: Cadherin 8, type 2
HPV: Human papillomavirus
hrHPV: High-risk human papillomavirus
HSIL: High-grade squamous intraepithelial lesion
IARC: International Agency for Research on Cancer
LSIL: Low-grade squamous intraepithelial lesion
*MGMT*: O-6-methylguanine-DNA methyltransferase
NILM: Negative for intraepithelial lesion or malignancy
Pap: Papanicolaou smear
ROC: Receiver operating characteristic
TCGA: The Cancer Genome Atlas
*ZNF582*: Zinc finger protein 582
