# Supplementary material for: Molecular Pap smear: HPV genotype and DNA methylation of ADCY8, CDH8, and ZNF582 as an integrated biomarker for high-grade cervical cytology
Source: Clin Epigenetics. 2016 Sep 13;8(1):96. doi: 10.1186/s13148-016-0263-9 (PMC5022163; doi:10.1186/s13148-016-0263-9)
Supplement: Additional file 1: Table S1. — Genes screened for promoter methylation in cervical cytology samples [48–52]. (PDF 132 kb) [file 13148_2016_263_MOESM1_ESM.pdf]

Supplementary Table S1 Genes screened for promoter methylation in cervical cytology samples

Gene	Assay[a]	Gene name	Chr	Gene-encoded protein function
ADCY8	ADCY8_a1	adenylate cyclase 8 (brain)	8	Membrane-bound enzyme that catalyzes the formation of cyclic AMP from ATP.
ADRA1A	ADRA1A_a1	adrenergic receptor Alpha-1A	8	G protein-coupled receptor that activates mitogenic responses and regulates the growth and proliferation of many cells.
AKT1	AKT1_01	v-akt murine thymoma viral oncogene homolog 1	14	Serine-threonine protein kinase that regulates many processes including metabolism, proliferation, cell survival, growth and angiogenesis.
AKT3	AKT3_01	v-akt murine thymoma viral oncogene homolog 3	1	Serine-threonine protein kinase that regulates many processes including metabolism, proliferation, cell survival, growth and angiogenesis.
ALK	ALK_a5, _a7[b]	anaplastic lymphoma receptor tyrosine kinase	2	Receptor tyrosine kinase belonging to the insulin receptor superfamily.
APC	APC_a1, _a3	adenomatous polyposis coli	5	Tumor suppressor protein that acts as an antagonist of the Wnt signaling pathway and is also involved in cell migration, adhesion, transcriptional activation, and apoptosis.
BCAR3	BCAR3_03	breast cancer anti-estrogen resistance 3	1	Component of intracellular signal transduction that causes estrogen-independent proliferation in human breast cancer cells.
BMP2	BMP2_01	bone morphogenetic protein 2	20	Transforming growth factor-beta (TGFB) superfamily.
BMP3	BMP3_01, _02	bone morphogenetic protein 3	4	Transforming growth factor-beta (TGFB) superfamily.
BMP4	BMP4_01	bone morphogenetic protein 4	14	Transforming growth factor-beta (TGFB) superfamily.
BMP7	BMP7_01	bone morphogenetic protein 7	20	Transforming growth factor-beta (TGFB) superfamily.

CCNA1	CCNA1_01	cyclin A1	13	Cyclin that binds to important cell cycle regulators, such as Rb family proteins, transcription factor E2F-1, and the p21 family proteins.
CDH8	CDH8_01	cadherin 8, type 2	16	Type II cadherin integral membrane protein that mediates calcium-dependent cell-cell adhesion.
CDH13	CDH13_yh1	cadherin 13	16	Cadherin localized on the cell membrane that is anchored by a GPI moiety, rather than by a transmembrane domain. This gene is hypermethylated in many types of cancers.
CDKN2A	CDKN2A_01,_02	cyclin-dependent kinase inhibitor 2A	9	Isoforms function as inhibitors of CDK4 kinase or stabilizers of the tumor suppressor protein p53. This gene is mutated or deleted in many types of cancers.
Chr3_a1	Chr3_a1	intergenic region	3	DMR in oral, endometrial and breast cancers.[c]
Chr3_a2	Chr3_a2	intergenic region	3	DMR in oral, endometrial and breast cancers.[c]
Chr7_a1	Chr7_a1	intergenic region	3	DMR in oral, endometrial and breast cancers.[c]
Chr7_a2	Chr7_a2	intergenic region	3	DMR in oral, endometrial and breast cancers.[c]
Chr7_a3	Chr7_a3	intergenic region	3	DMR in oral, endometrial and breast cancers.[c]
Chr7_a4	Chr7_a4	intergenic region	3	DMR in oral, endometrial and breast cancers[c]
Chr8_a1	Chr8_a1	intergenic region	3	DMR in oral, endometrial and breast cancers.[c]
CMTM1	CMTM1_01	CKLF-like MARVEL transmembrane domain containing 1	16	Chemokine and the 4 transmembrane superfamilies of signaling molecules.

DAPK1	DAPK1_01	death-associated protein kinase 1	9	Serine-threonine kinase that mediates gamma-interferon-induced programmed cell death.
EPHA5	EPHA5_01	EPH receptor A5	4	Ephrin receptor subfamily of the protein-tyrosine kinase family.
EPHB1	EPHB1_01,_02	EPH receptor B1	3	Ephrin receptor subfamily of the protein-tyrosine kinase family.
EPHB3	EPHB3_04,_05	EPH receptor B3	3	Ephrin receptor subfamily of the protein-tyrosine kinase family.
FOXA1	FOXA1_a1,_a2,_a3[d]	forkhead box A1	14	Forkhead class of DNA-binding proteins that function as a hepatocyte transcriptional activator.
FOXB2	FOXB2_a2_02[d]	forkhead box AB2	9	Forkhead class of DNA-binding proteins.
FOXE1	FOXE1_01	forkhead box E1	9	Forkhead class of DNA-binding proteins that functions as a thyroid transcription factor.
GSTP1	GSTP1_a1	glutathione S-transferase pi 1	11	Glutathione S-transferases (GSTs) are enzymes that detoxify hydrophobic and electrophilic compounds. They play a role in susceptibility to cancer.
HIF1A	HIF1A_01	Hypoxia-inducible factor 1, alpha subunit	14	Alpha subunit of the transcription factor hypoxia-inducible factor-1 (HIF-1), which acts as a master regulator of cellular and systemic homeostatic responses to hypoxia by activating the transcription of many genes.
IRS1	IRS1_05	insulin receptor substrate 1	2	Cellular protein that is phosphorylated by insulin receptor tyrosine kinase and mediates the control of various cellular processes by insulin.
KDM6B	KDM6B_a2,_a3	lysine (K)-specific demethylase 6B	17	Histone demethylase that specifically demethylates 'Lys-27' of histone H3, thereby catalyzing the removal of methyl groups from lysine and arginine residues on histone tails.
L1-*MET*	L1-MET_a1	LINE-1 MET<br>Long interspersed nuclear elements MET proto-oncogene, receptor tyrosine kinase	7	A specific LINE 1 located within the *MET* oncogene.[e]

LINE1	LINE1_a1 sup[e]	Long interspersed nuclear elements	22	Group of genetic elements found in large numbers in eukaryotic genomes.
MGMT	MGMT_01	O-6-methylguanine-DNA methyltransferase	10	O-6-methylguanine-DNA methyltransferase, an enzyme involved in DNA repair that removes alkylating lesions at the O6 of guanine residues.
NEFL	NEFL_a1cm	neurofilament, light polypeptide	8	Neurofilaments comprise the exoskeleton that maintain the neuronal caliber and intracellular transport to axons and dendrites.
OGG1	OGG1_03	8-oxoguanine DNA glycosylase	3	Glycosylase enzyme responsible for excising 8-oxoguanine, a mutagenic base byproduct that occurs as a result of exposure to reactive oxygen.
RARB	RARB_a1	retinoic acid receptor, beta	3	Retinoic acid receptor beta binds retinoic acid, the biologically active form of vitamin A, which mediates cellular signaling.
RASSF1	RASSF1_01,_02,_03	Ras association domain family member 1	3	Protein similar to the RAS effector proteins with a tumor suppressor function that is required for death receptor-dependent apoptosis.
SNAI 1	SNAI 1_01	snail family zinc finger 1	20	Snail nuclear protein is a zinc finger transcriptional repressor that down-regulates the expression of ectodermal genes within the mesoderm.
TCF3_01	TCF3_01	transcription factor 3	19	E protein (class I) family of helix-loop-helix transcription factors involved in the initiation of neuronal differentiation.
TSC2	TSC2_02	tuberous sclerosis 2	16	Tumor suppressor protein that stimulates specific GTPases. This protein associates with hamartin in a cytosolic complex, possibly acting as a hamartin chaperone.
TWIST 1	TWIST 1_01	twist family bHLH transcription factor 1	7	Basic helix-loop-helix (bHLH) transcription factor implicated in cell lineage determination and differentiation.
VEGFA	VEGFA_01	vascular endothelial growth factor A	6	Glycosylated mitogen that specifically acts on endothelial cells to mediate vascular permeability, induce angiogenesis and vasculogenesis, promote cell migration, and inhibit apoptosis.
VHL	VHL_01	von Hippel-Lindau tumor suppressor, E3 ubiquitin protein ligase	3	Component of a protein complex involved in ubiquitination and the degradation of hypoxia-inducible-factor (HIF), a transcription factor that regulates gene expression by oxygen.

ZNF582	ZNF582_01	zinc finger protein 582	19	Zinc finger protein involved in transcriptional regulation.

Chr, chromosome; DMR, differentially methylated region from MBDcap-seq whole genome screening of 1000 methylomes from http://cbbiweb.uthscsa.edu/kMethylationWS/ws.

[a]Assays were designed in-house (alphanumerical suffix) or acquired commercially from QIAGEN (numerical suffix). See Table S2 for assay details.

[b]Reference #48.

[c]Reference #48-50.

[d]Reference #51.

[e]Reference #52.
