# Supplementary material for: Molecular Pap smear: HPV genotype and DNA methylation of ADCY8, CDH8, and ZNF582 as an integrated biomarker for high-grade cervical cytology
Source: Clin Epigenetics. 2016 Sep 13;8(1):96. doi: 10.1186/s13148-016-0263-9 (PMC5022163; doi:10.1186/s13148-016-0263-9)
Supplement: Additional file 2: Table S2. — List of pyrosequencing assays. (PDF 186 kb) [file 13148_2016_263_MOESM2_ESM.pdf]

Supplementary Table S2 List of pyrosequencing assays

Assay[a]	Primer[b]	Sequence 5' to 3'
ADCY8_a1	Forward<br>Reverse<br>Sequencing	AGTGGAGGTTAGGGTAAAGTTT<br>Biotin-TAACCCCTCCATAACTATAACCCCCATTAA<br>GGGAGGAGGTTTTAATTATT
ADRA1A_a1	Forward<br>Reverse<br>Sequencing	GGAATTGTATGTTGTAAGGAGTTT<br>Biotin-ACAACCCTAACCAACCCTAAAA<br>TGTAAGGAGTTTTTTGGA
AKT1_01		Qiagen Hs_BMP3_01 PM00112595
AKT3_01		Qiagen Hs_AKT2_01 PM00086044
ALK_a5	Forward<br>Reverse<br>Sequencing	AGTTGGGGGAAAGTAAGAGA<br>Biotin-AAAAACTCTAAACAATCCTTAATACC<br>GGGGAAAGTAAGAGAT
ALK_a7	Forward<br>Reverse<br>Sequencing	GGTAGAGGTGGTAGTAGTTGGTATT<br>Biotin-CCCTCCTCACCCATCATCA<br>GTGGTAGTAGTTGGTATTT
APC_a1	Forward<br>Reverse<br>Sequencing	GGTATGGGGTTAGGGTTAGGTAGG<br>Biotin-AATCCCACAACACCTCCATTCTATCT<br>AGAGAAGTAGTTGTGTAAT
APC_a3	Forward<br>Reverse<br>Sequencing	GGGTTAGGGTTAGGTAGGTTGT<br>Biotin-AATTACACAACTACTTCTCTCC<br>GGTTAGGGTTAGGTAGGTT
BCAR3_03		Qiagen HS_BCAR3_03 PM00088326
BMP2_01		Qiagen HS_BMP2_01 PM00196763
BMP3_01		Qiagen Hs_BMP3_01 PM00112595
BMP3_02		Qiagen HS_BMP3_02 PM00112602
BMP4_01		Qiagen HS_BMP4_01 PM00056525
BMP7_01		Qiagen Hs_BMP7_01 PM00079345
CCNA1_01		Qiagen Hs_CCNA1_01 PM00162708

CDH8_01		Qiagen HS_CDH8_01 PM00062944
CDH13_yh1	Forward<br>Reverse<br>Sequencing	Biotin-AGTTTAAAGAAGTAAATGGGATGTTAT<br>TCTACCTCCCCAACCAACCAACTTC<br>ACCAAAACCAATAACTTTAC
CDKN2A_01		Qiagen Hs_CDKN2A_01 PM00039900
CDKN2A_02		Qiagen Hs_CDKN2A_02 PM00039907
Chr3_a1	Forward<br>Reverse<br>Sequencing	Biotin-AAGAGATGGGGTTTTTAGGAAGTG<br>CACCATTCAACATCTAACAAAAACTTAAA<br>AATCATTTTTACTTTAAAAATAATA
Chr3_a2	Forward<br>Reverse<br>Sequencing	GAGTTTTTATGAATGGGATTGGTGTTT<br>Biotin-AAACCTCTAACACCTACAACTAC<br>GAGTTTATTTATTTTTTTTGTTATG
Chr7_a1	Forward<br>Reverse<br>Sequencing	TGTTTTTTGTGTTTTTGGTTTTTGTTAA<br>Biotin-AATAACCAATACAAAACACTTTCCCTTACT<br>GTTAAGTTTTTTTGTTTTGGG
Chr7_a2	Forward<br>Reverse<br>Sequencing	TTGAGGGAGTAAGGGAAAGTG<br>Biotin-ACTCTTAAAATTTCCTAACACAACTATC<br>GAGTAAGGGAAAGTGT
Chr7_a3	Forward<br>Reverse<br>Sequencing	GGAGGAGTTTTTGGAGTAGG<br>Biotin-ACCCTCAACACCAACAAA<br>TGTTAGGAAATTTTAAGAGTATT
Chr7_a4	Forward<br>Reverse<br>Sequencing	GTGTTGAGGGTTTTTGAGATTAT<br>Biotin-ATCCTCCTAACTAAATAAAACTACCTCACT<br>TTTGTTTAGAGTTTTTTAAG
Chr8_a1	Forward<br>Reverse<br>Sequencing	TTGGTTTAGAAGTTTTTTTATTGAGTATT<br>Biotin-ATCCCCTAATCCACCAAATTTCATA<br>AGTTTTTTTATTGAGTATTTTGTG
CMTM1		Qiagen Hs_CMTM1_01 PM00061215
DAPK1_01		Qiagen Hs_DAPK1_01 PM00041258
EPHA5_01		Qiagen Hs_EPHA5_01 PM00018683
EPHB1_01		Qiagen Hs_EPHB1_01 PM00015183
EPHB1_02		Qiagen_Hs_EPHB1_02 PM00015190

EPHB3_04		Qiagen Hs_EPHB3_04 PM00109641
EPHB3_05		Qiagen Hs_EPHB3_05 PM00109648
FHIT_01		Qiagen Hs_FHIT_01 PM00017129
FOXA1_a1	Forward<br>Reverse<br>Sequencing	GGATAATAAAGTTATTTGTTGGGAAATAGA<br>Biotin-CTTACACCCCAAACTCAAAAACTAACTTC<br>ATGTTTATAGGTGATTTGTTTTA
FOXA1_a2	Forward<br>Reverse<br>Sequencing	GAGGGGGTTTTGGAAGTT<br>Biotin-CTAACCCCAAAACAAATACAAAACACTTT<br>GGGTTTTGGAAGTTAGT
FOXA1_a3	Forward<br>Reverse<br>Sequencing	ATTTTTTTGGGGTTATATTTTTTGTGAG<br>Biotin- ACCCAAACCTAAATTCATATTACTAAC<br>TTTTTTTTGTAGTGTAAGATAT
FOXB2_a2	Forward<br>Reverse<br>Sequencing	GAGTATATATAGAGTTGGTAGAATAGTT<br>Biotin- ATAATAAAAATAAAAATACCCTCCC<br>AGAATAGTTTGAGTTATAATTTTTT
FOXB2_02		Qiagen Hs_CDKN2A_02 PM00039907
FOXE1_01		Qiagen Hs_FOXE1_01 PM00041013
GSTP1_a1	Forward<br>Reverse<br>Sequencing	GAGTTAGAGGGATTTTTTAGAAGAG<br>CAATTAACCCCATACTAAAAACTCT<br>GGAGAGGGGAGGGATTATTTTTATA
HIF1A_01		Qiagen Hs_HIF1A PM00056147
IRS1_05		Qiagen Hs_IRS1_05 PM00011711
KDM6B_a2	Forward<br>Reverse<br>Sequencing	TAGGGAGTTTTGAGGATAAGTAGGG<br>Biotin-CACATACCCTTCTATATTTACCTTCATATT<br>GAAGGTAGATAGATTTTTAAATTTT
KDM6B_a3	Forward<br>Reverse<br>Sequencing	GTTATAGGGAGTTTTGAGGATAAGT<br>Biotin-ACATACCCTTCTATATTTACCTTCATATT<br>GAGGAGTGTAGTAAGGA
L1-MET_a1	Forward<br>Reverse<br>Sequencing	ATTGTTTGGTATTTTTTAGTGAGATGAA<br>Biotin-ATCATAATCCTCCAACCACTAC<br>ATTTTAGATGGAAATGTAGAAA
LINE 1_a1sup	Forward<br>Reverse<br>Sequencing	TTGGGAAGAGTAAGGGGTTAGG<br>Biotin-ACCCTCTAAACCAAATATAAAATATAAT<br>GAAAATCGGGTTATTTT

MGMT		Qiagen Hs_MGMT_01 PM00149702
NEFL	Forward<br>Reverse<br>Sequencing	AGTTGGAGTAGTAGAATAAGGT<br>Biotin-ATTACCCAAAATCTCCTCCAACCCTTC<br>TTGGTGTTGAGTTAGAAGTAT
OGG1_03		Qiagen Hs_OGG1_03 PM00013776
RARB_a1	Forward<br>Reverse<br>Sequencing	TTGTTAAAGGGGGGATTAGAAT<br>Biotin-ACCCAAACAAACCCTACT<br>GTTTGAGGATTGGGAT
RASSF1_01		Qiagen Hs_RASSF1_01 PM00013292
RASSF1_02		Qiagen Hs_RASSF1_02 PM00013300
RASSF1_03		Qiagen Hs_RASSF1_03 PM00013307
SNAI1_01		Qiagen Hs_SNAI1_01 PM00196588
TCF3_01		Qiagen Hs_TCF3_01 PM00072219
TSC2_02		Qiagen Hs_TSC2_02 PM00172886
TWIST 1_01		Qiagen Hs_TWIST_01_ PM00030121
VEGFA_01		Qiagen Hs_VEGFA_01 PM00121968
VHL_01		Qiagen Hs_VHL_01 PM00014504
ZNF582_01		Qiagen Hs_ZNF582_01 PM00071925

[a]Assays were designed in-house (alphanumerical suffix) or acquired commercially from QIAGEN (numerical suffix).

[b]Types of in-house designed primers.
