# Supplementary material for: Molecular Pap smear: HPV genotype and DNA methylation of ADCY8, CDH8, and ZNF582 as an integrated biomarker for high-grade cervical cytology
Source: Clin Epigenetics. 2016 Sep 13;8(1):96. doi: 10.1186/s13148-016-0263-9 (PMC5022163; doi:10.1186/s13148-016-0263-9)
Supplement: Additional file 3: Figure S1. — DNA methylation and gene expression of ADCY8, CDH8, ZNF582, and MGMT in the TCGA cervical cancer cohort. (PDF 106 kb) [file 13148_2016_263_MOESM3_ESM.pdf]

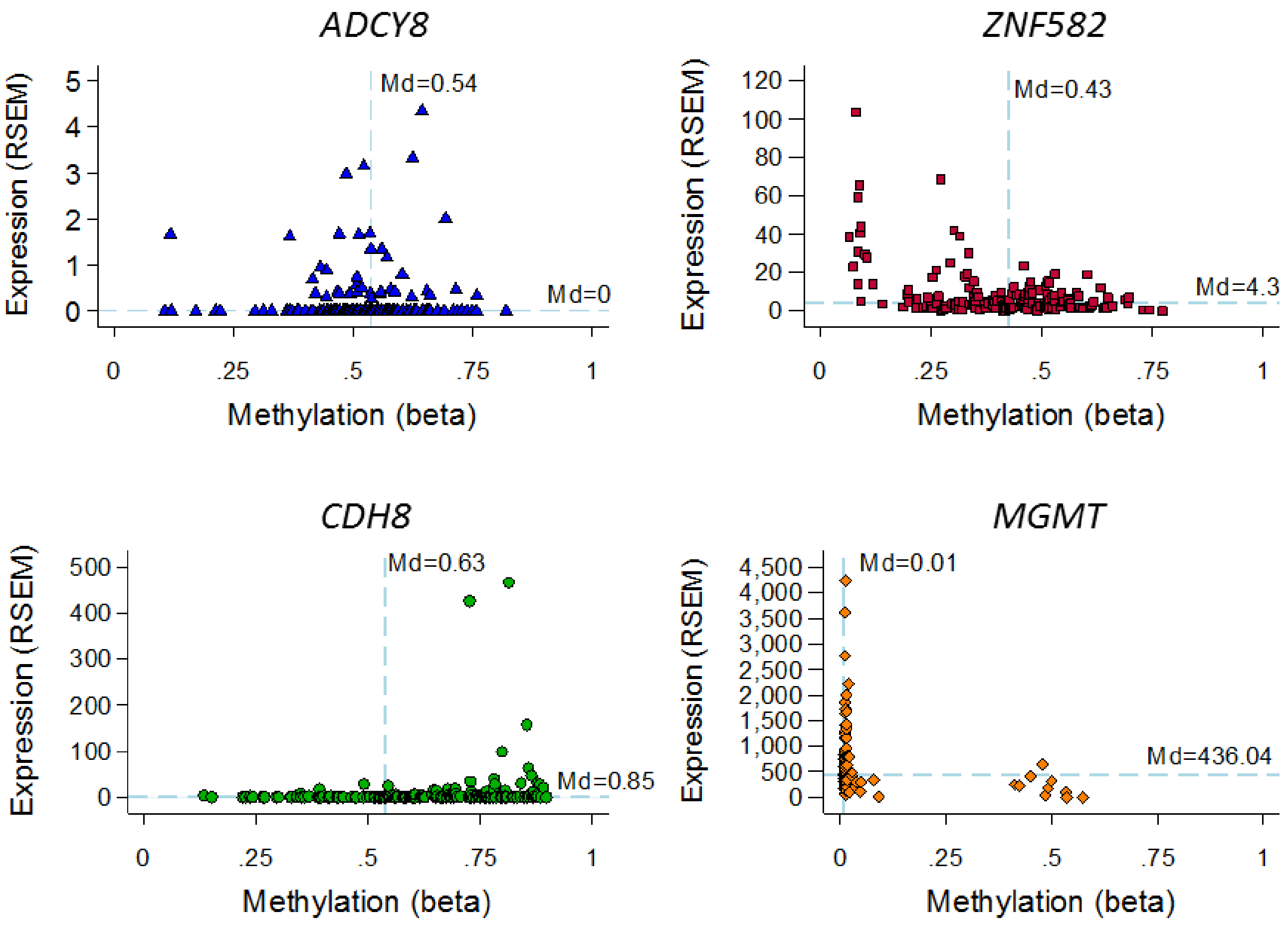

Supplementary Figure S1. DNA methylation and gene expression of *ADCY8*, *CDH8*, *ZNF582*, and *MGMT* in the TCGA cervical cancer cohort. Correlation plot for 181 patient tumors using paired median CpG methylation (beta value) and expression value (RSEM-normalized) from each sample. Median values (Md) for each axis are marked with a dotted line. (*ADCY8*: rho = 0.01, p = 0.94; *ZNF582*: rho = -0.23, p = 0.001; *CDH8*: rho = 0.27, $p < 0.001$; *MGMT*: rho = -0.17, p = 0.03).
