# Supplementary material for: Molecular Pap smear: HPV genotype and DNA methylation of ADCY8, CDH8, and ZNF582 as an integrated biomarker for high-grade cervical cytology
Source: Clin Epigenetics. 2016 Sep 13;8(1):96. doi: 10.1186/s13148-016-0263-9 (PMC5022163; doi:10.1186/s13148-016-0263-9)
Supplement: Additional file 4: Table S3. — Logistic regression analysis of HPV and HPV + ZNF582 for predicting abnormal (LSIL/HSIL) cytology. (PDF 151 kb) [file 13148_2016_263_MOESM4_ESM.pdf]

Supplementary Table S3 Logistic regression analysis of HPV and HPV + *ZNF582* for predicting abnormal (LSIL/HSIL) cytology

Variable	Coefficient (β)	SE	z	P>z	OR	95% CI		
				Univariable model				
HPV[a]	1.77	0.27	6.50	0.00	5.86	3.44	to	9.99
constant	-1.66	0.47	-3.52	0.00	0.19	0.08	to	0.48
				Multivariable model				
HPV[a]	1.80	0.31	5.84	0.00	6.04	3.30	to	11.04
ZNF582[b]	2.69	0.88	3.05	0.00	14.72	2.61	to	83.06
constant	-2.51	0.66	-3.80	0.00	0.08	0.02	to	0.30

HPV, human papillomavirus; HSIL, high-grade squamous intraepithelial lesion; LSIL, low-grade squamous intraepithelial lesion; ZNF, *ZNF582* gene; SE, standard error; z, z-score.

[a]The HPV genotype identified in each sample was coded accordingly: HPV undetected (0), not classifiable (1), possibly carcinogenic (2), and carcinogenic (3).

[b]The quantified promoter methylation value (%) of *ZNF582* gene at CpG-position 3 of each sample was binarized accordingly: <1.1 (0), ≥1.1 (1).
