# Supplementary material for: Molecular Pap smear: HPV genotype and DNA methylation of ADCY8, CDH8, and ZNF582 as an integrated biomarker for high-grade cervical cytology
Source: Clin Epigenetics. 2016 Sep 13;8(1):96. doi: 10.1186/s13148-016-0263-9 (PMC5022163; doi:10.1186/s13148-016-0263-9)
Supplement: Additional file 5: Table S4. — Logistic regression analysis of HPV and HPV + three-gene methylation markers for predicting for HSIL cytology. (PDF 154 kb) [file 13148_2016_263_MOESM5_ESM.pdf]

Supplementary Table S4 Logistic regression analysis of HPV and HPV + 3-gene methylation markers for predicting HSIL cytology

Variable	Coefficient (β)	SE	z	P>z	OR	95% CI		
Univariable model								
HPV[a]	1.41	0.32	4.42	0.00	4.09	2.19	to	7.65
constant	-3.95	0.90	-4.37	0.00	0.02	0.00	to	0.11
Multivariable model								
HPV[a]	1.55	0.37	4.19	0.00	4.72	2.28	to	9.74
ADCY8[b]	1.16	0.48	2.40	0.02	3.17	1.24	to	8.15
CDH8[c]	1.04	0.46	2.26	0.02	2.82	1.15	to	6.95
ZNF582[d]	1.36	0.46	2.98	0.00	3.91	1.59	to	9.59
constant	-5.78	1.13	-5.12	0.00	0.00	0.00	to	0.03

HPV, human papillomavirus; HSIL, high-grade squamous intraepithelial lesion; LSIL, low-grade squamous intraepithelial lesion; ZNF, *ZNF582* gene; SE, standard error; z, z-score.

[a]The HPV genotype identified in each sample was coded accordingly: HPV undetected (0), not classifiable (1), possibly carcinogenic (2), and carcinogenic (3).

[b]The quantified promoter methylation value (%) of *ADCY8* gene at CpG-position 7 of each sample was binarized accordingly: <5.8 (0), ≥5.8 (1).

[c]The quantified promoter methylation value (%) of *CDH8* gene at CpG-position 3 of each sample was binarized accordingly: <3.0 (0), ≥3.0 (1).

[d]The quantified promoter methylation value (%) of *ZNF582* gene at CpG-position 3 of each sample was binarized accordingly: <1.1 (0), ≥1.1 (1).
