# Supplementary material for: Molecular Pap smear: HPV genotype and DNA methylation of ADCY8, CDH8, and ZNF582 as an integrated biomarker for high-grade cervical cytology
Source: Clin Epigenetics. 2016 Sep 13;8(1):96. doi: 10.1186/s13148-016-0263-9 (PMC5022163; doi:10.1186/s13148-016-0263-9)
Supplement: Additional file 7: Table S6. — Predictive margins for HSIL cytology based on HPV or HPV + three-gene methylation markers. (PDF 196 kb) [file 13148_2016_263_MOESM7_ESM.pdf]

Supplementary Table S6 Predictive margins for HSIL cytology based on HPV or HPV + 3-gene methylation markers

	MER	Predictors				Predicted Probability Statistics						
Outcome	at	HPV[a]	ADCY[b]	CDH[b]	ZNF[b]	Margin[c]	SE	z	P>z	95% CI		
Univariable logit model												
NILM/LSIL (0), HSIL (1)	1	0	-	-	-	0.02	0.02	1.13	0.26	-0.01	to	0.05
	2	1	-	-	-	0.07	0.04	1.81	0.07	-0.01	to	0.15
	3	2	-	-	-	0.24	0.06	4.30	0.00	0.13	to	0.36
	4	3	-	-	-	**0.57**[d]	0.05	11.71	0.00	0.47	to	0.67
Multivariable logit model												
NILM/LSIL (0), HSIL (1)	1	0	0	0	0	0.00	0.00	0.89	0.37	0.00	to	0.01
	2	0	0	0	1	0.01	0.01	0.93	0.35	-0.01	to	0.04
	3	0	0	1	0	0.01	0.01	0.89	0.37	-0.01	to	0.03
	4	0	0	1	1	0.03	0.03	0.96	0.34	-0.03	to	0.10
	5	0	1	0	0	0.01	0.01	0.88	0.38	-0.01	to	0.03
	6	0	1	0	1	0.04	0.04	0.97	0.33	-0.04	to	0.11
	7	0	1	1	0	0.03	0.03	0.92	0.36	-0.03	to	0.08
	8	0	1	1	1	0.10	0.09	1.09	0.28	-0.08	to	0.27
	9	1	0	0	0	0.01	0.01	1.30	0.20	-0.01	to	0.04
	10	1	0	0	1	0.05	0.04	1.39	0.16	-0.02	to	0.13
	11	1	0	1	0	0.04	0.03	1.30	0.20	-0.02	to	0.10
	12	1	0	1	1	0.14	0.09	1.51	0.13	-0.04	to	0.32
	13	1	1	0	0	0.04	0.04	1.25	0.21	-0.02	to	0.11
	14	1	1	0	1	0.15	0.10	1.56	0.12	-0.04	to	0.35
	15	1	1	1	0	0.12	0.08	1.41	0.16	-0.05	to	0.28
	16	1	1	1	1	0.34	0.16	2.13	0.03	0.03	to	0.65
	17	2	0	0	0	0.06	0.03	2.25	0.02	0.01	to	0.12
	18	2	0	0	1	0.21	0.08	2.55	0.01	0.05	to	0.38
	19	2	0	1	0	0.16	0.07	2.22	0.03	0.02	to	0.31
	20	2	0	1	1	0.43	0.13	3.25	0.00	0.17	to	0.69
	21	2	1	0	0	0.18	0.09	2.08	0.04	0.01	to	0.35
	22	2	1	0	1	0.46	0.13	3.49	0.00	0.20	to	0.72
	23	2	1	1	0	0.38	0.13	2.85	0.00	0.12	to	0.64
	24	2	1	1	1	0.71	0.10	6.94	0.00	0.51	to	0.91
	25	3	0	0	0	0.25	0.06	3.94	0.00	0.12	to	0.37
	26	3	0	0	1	0.56	0.11	5.13	0.00	0.35	to	0.77
	27	3	0	1	0	**0.48**[d]	0.11	4.20	0.00	0.26	to	0.70
	28	3	0	1	1	0.78	0.09	8.79	0.00	0.61	to	0.96
	29	3	1	0	0	0.51	0.13	3.95	0.00	0.26	to	0.76
	30	3	1	0	1	0.80	0.08	9.79	0.00	0.64	to	0.96

MER	Predictors				Predicted Probability Statistics						
at	HPV[a]	ADCY[b]	CDH[b]	ZNF[b]	Margin[c]	SE	z	P>z	95% CI		
31	3	1	1	0	0.74	0.10	7.47	0.00	0.55	to	0.94
32	3	1	1	1	0.92	0.04	24.54	0.00	0.85	to	0.99

3-gene methylation markers (*ADCY8, CDH8, ZNF582)*; ADCY, *ADCY8* gene; at, number of possible combinations of representative values from each predictor variable; CDH, *CDH8* gene; HPV, human papillomavirus; HSIL, high-grade squamous intraepithelial lesion; LSIL, low-grade squamous intraepithelial lesion; MER, marginal effect at representative values (a statistical method to calculate margins); NILM, negative for intraepithelial lesion/malignancy; ZNF, *ZNF582* gene; SE, standard error; z, z-score.

[a]The HPV genotype identified in each sample was coded accordingly: HPV undetected (0), not classifiable (1), possibly carcinogenic (2), and carcinogenic (3).

[b]The quantified promoter methylation value (%) of *ADCY8* at CpG-position 7, *CDH8* at CpG-position 3, and *ZNF582* at CpG-position 3 of each sample was binarized accordingly: <5.8 (0), ≥5.8 (1); <3.0 (0), ≥3.0 (1); <1.1 (0), ≥1.1 (1).

[c]The margin is the predicted probability for the outcome of interest based on representative values from each predictor variable.

[d]The margins (in bold) are the positive outcome or “classification threshold” probabilities used for classification of outcomes and the evaluation of diagnostic test performance. The classification threshold is estimated by using the maximum sum of sensitivity and specificity (Youden’s index).
