# Supplementary material for: Molecular Pap smear: HPV genotype and DNA methylation of ADCY8, CDH8, and ZNF582 as an integrated biomarker for high-grade cervical cytology
Source: Clin Epigenetics. 2016 Sep 13;8(1):96. doi: 10.1186/s13148-016-0263-9 (PMC5022163; doi:10.1186/s13148-016-0263-9)
Supplement: Additional file 8: Table S7. — Diagnostic performance of HPV vs. HPV + ZNF582 for abnormal (LSIL/HSIL) cytology. (PDF 152 kb) [file 13148_2016_263_MOESM8_ESM.pdf]

Supplementary Table S7 Diagnostic performance of HPV vs. HPV + *ZNF582* for abnormal (LSIL/HSIL) cytology

	Biomarkers	
Parameters	HPV	HPV + *ZNF582*
Disease (cytological)[a]		
Present (LSIL/HSIL)	125 (80)	125 (80)
Absent (NILM)	31 (20)	31 (20)
Total	156 (100)	156 (100)
Diagnostic performance[b, c]		
Sensitivity	112/125 (90)	125/125 (100)
Specificity	25/31 (81)	21/31 (68)
PPV	112/118 (95)	125/135 (93)
NPV	25/38 (66)	21/21 (100)
False positive	6/118 (5)	10/135 (7)
False negative	13/38 (34)	0/21 (0)
LR + test (TP/FP)	4.74	3.13
LR - test (FN/TN)	0.123	0
Accuracy, %	137/156 (88)	146/156 (94)

FN, false negative; FP, false positive; HPV, human papillomavirus; HSIL, high-grade squamous intraepithelial lesion; LR, likelihood ratio; LSIL, low-grade squamous intraepithelial lesion; NILM, negative for intraepithelial lesion/malignancy; TN, true negative; TP, true positive; ZNF, *ZNF582* gene.

[a]The disease state was defined by the cytological diagnosis of the specimen at study entry. It served as the reference standard for the clinical performance of the molecular tests, i.e., HPV and HPV + *ZNF582.*

[b]The positive outcome or “classification threshold” probabilities used for classification of outcomes for HPV and HPV + *ZNF582* were 0.53 and 0.33, respectively.

[c]Values are n/N (%) unless denoted otherwise.
