# Supplementary material for: Molecular Pap smear: HPV genotype and DNA methylation of ADCY8, CDH8, and ZNF582 as an integrated biomarker for high-grade cervical cytology
Source: Clin Epigenetics. 2016 Sep 13;8(1):96. doi: 10.1186/s13148-016-0263-9 (PMC5022163; doi:10.1186/s13148-016-0263-9)
Supplement: Additional file 9: Table S8. — Diagnostic performance of HPV vs. HPV + three-gene methylation markers for HSIL cytology. (PDF 152 kb) [file 13148_2016_263_MOESM9_ESM.pdf]

Supplementary Table S8 Diagnostic performance of HPV vs. HPV + 3-gene methylation markers for HSIL cytology

Parameters	Biomarkers	
	HPV	HPV + *ADCY8* + *CDH8* + *ZNF582*
Disease (cytological)[a]		
Present (HSIL)	63 (40)	63 (40)
Absent (NILM/LSIL)	93 (60)	93 (60)
Total	156 (100)	156 (100)
Diagnostic performance[b, c]		
Sensitivity	58/63 (92)	48/63 (76)
Specificity	51/93 (55)	82/93 (88)
PPV	58/100 (58)	48/59 (81)
NPV	51/56 (91)	82/97 (85)
False positive	42/100 (42)	11/59 (19)
False negative	5/56 (9)	15/97 (15)
LR + test (TP/FP)	2.04	6.33
LR - test (FN/TN)	0.15	0.27
Accuracy, %	109/156 (70)	130/156 (83)

ADCY, *ADCY8* gene; CDH, *CDH8* gene; FN, false negative; FP, false positive; HPV, human papillomavirus; HSIL, high-grade squamous intraepithelial lesion; LR, likelihood ratio; LSIL, low-grade squamous intraepithelial lesion; NILM, negative for intraepithelial lesion/malignancy; TN, true negative; TP, true positive; ZNF, *ZNF582* gene.

[a]The disease state was defined by the cytological diagnosis of the specimen at study entry. It served as the reference standard for the clinical performance of the molecular tests i.e., HPV and HPV + 3-gene methylation markers.

[b]The positive outcome or “classification threshold” probabilities used for classification of outcomes for HPV and HPV + 3-gene methylation markers were 0.57 and 0.48, respectively.

[c]Values are n/N (%) unless denoted otherwise.
